# Surveillance of TB-HIV coinfection in Brazil: a space-time approach

**DOI:** 10.1590/1980-549720240037

**Published:** 2024-07-15

**Authors:** Beatriz Almeida Santos, Caíque Jordan Nunes Ribeiro, Allan Dantas dos Santos, Álvaro Francisco Lopes de Sousa, Thayane Santos Siqueira, Lucas Almeida Andrade, Adriano José dos Santos, Shirley Verônica Melo Almeida Lima

**Affiliations:** I Universidade Federal de Sergipe, Graduate Program in Nursing – São Cristóvão (SE), Brazil.; II Universidade Nova de Lisboa, Institute of Hygiene and Tropical Medicine – Lisbon, Portugal.; III Universidade Federal de Sergipe, Graduate Program in Health Sciences – São Cristóvão (SE), Brazil.; IV Universidade Federal de Alagoas – Arapiraca (AL), Brazil.

**Keywords:** Tuberculosis, HIV, Time series studies, Spatial analysis

## Abstract

**Objective:**

To identify the epidemiological, spatial, and temporal pattern of TB-HIV coinfection in Brazil during the period from 2001 to 2020.

**Methods:**

Ecological study using space-time analysis techniques. It included cases of TB-HIV coinfection registered in Brazil from 2001 to 2020. The temporal trend analysis was performed using segmented regression by Joinpoint regression. For spatial analysis, Moran indices were calculated and choropleth maps were produced using TerraView and QGIS software.

**Results:**

A stable temporal trend was observed in the incidence rates of TB-HIV coinfection in Brazil during the analyzed period. In addition, high-risk areas for coinfection located in states in the North, Southeast, South, and Midwest regions were identified.

**Conclusion:**

There was stability in the incidence of TB-HIV coinfection in Brazil over the last 20 years and heterogeneous geographic distribution of risk areas for the condition.

## INTRODUCTION

Tuberculosis (TB) remains a global public health challenge and became even more challenging when its effects devastatingly impacted people living with Human Immunodeficiency Virus (HIV). The TB-HIV association causes important clinical complications and treatment adherence, showing that individuals with HIV are 28 times more likely to contract TB^1^. The high mortality, the increase in cases of drug-resistant TB, and the concentration of the disease in socially vulnerable populations encouraged the prioritization of combating the disease, whether at the global or national levels^2^.

Over the years, TB has remained stigmatized and has taken on different connotations in history, with emphasis on a reemerging characterization in some Western European and American countries^3,4^. In Brazil, it behaves as a serious public health issue involving multicentric, behavioral, and social factors. Globally, TB has caused 300 thousand deaths among HIV-positive people. Brazil is the only country in the Americas with priority for actions to control coinfection, as it represents 10.2% among those diagnosed with TB^5,6^.

To enable the global response, the World Health Organization (WHO) approved, at the 2014 World Health Assembly, the End TB Strategy^5^, which proposes a radical paradigm shift in the fight against TB, with the objective of eradicating the disease as a public health issue: reducing TB cases by 90% and deaths from TB by 95% until 2035^5^.

The global goals are underpinned by three pillars:

1. Integrated, patient-centered care and prevention;
2. Bold policies and support systems for those affected by TB; and
3. Intensification of innovation and research^5^.

The global emergency of the new coronavirus (COVID-19) pandemic culminated in the reorganization of healthcare actions, services, and systems throughout the world, and years of progress in controlling TB-HIV coinfection were reversed^1^. The pandemic process jeopardized the strategy to end TB in Brazil and directly impacted the new diagnoses^2^.

The COVID-19 pandemic exacerbated health inequalities and favored the neglect of TB-HIV coinfection from diagnosis to treatment^2^. From 2012 to 2019, the proportion of new TB cases tested for HIV increased by approximately 15%, reaching 82.8%, especially in 2019. However, in 2020 and 2021 there was an important decrease^4^. It is a fact that during the pandemic period there was a reduction in HIV testing in cases of TB.

In developing countries, such as Brazil, social inequality represents fertile ground for the spread of coinfection, considering that TB-HIV is associated with poor living, health, and work conditions. The WHO expressed concern about the possibility of compromising the progress achieved in relation to the prevention and treatment of TB, stressing the importance of national programs for the disease to ensure the continuity of actions aimed at controlling it^4,6^.

Knowing the temporal and spatial distribution, as well as identifying the profile of those affected, helps to guide policies for controlling TB-HIV, to direct efforts to areas with the highest risk, and to provide guidance for those where the operational situation of the National TB Control program falls short of the established goals. In this context, studies of this magnitude can contribute to the strengthening of new scientific investigations and the implementation of public policies aimed at confronting coinfection. Thus, our objective in this study is to identify the epidemiological, spatial, and temporal pattern of TB-HIV coinfection in Brazil during the period from 2001 to 2020.

## METHODS

### Study design

This is an ecological study using spatial analysis and temporal trend techniques. All confirmed cases of TB-HIV coinfection registered in Brazil between 2001 and 2020 were included, considering the months of January to December in every year, whose units of analysis and aggregation were the 5,570 Brazilian municipalities. Temporarily, the notified coinfection data linked to the Notifiable Diseases Information System (*Sistema de Informação de Agravos de Notificação* – SINAN), publicly available by the Department of Informatics of the Brazilian Unified Health System (*Departamento de Informática do Sistema Único de Saúde do Brasil* – DATASUS) in digital format, were collected. Thus, submission to the ethics committee was not required.

### Study area

Brazil covers an area of 8,510,345.540 million km², equivalent to almost 50% of the territory of South America, and has a total population of 215.1 million inhabitants^7^, being the largest country in Latin America and the sixth largest population in the world. Delimited by the Atlantic Ocean to the east, it has a coastline of 7,491 km and borders all other South American countries, except Chile and Ecuador. Brazil is divided into five regions (North, Northeast, Southeast, South, and Midwest) and 27 Federative Units (FU)^8^. According to the Human Development report (2021/2022^9^), it has a Human Development Index (HDI) of 0.754; therefore, it is within the high development range, despite having significant socioeconomic discrepancies between regions^10^.

### Exploratory data analysis

All new cases of TB-HIV coinfection registered in Brazil between 2001 and 2020 at SINAN were used as the outcome variable. The total number of cases of TB-HIV was divided by the population of new TB cases and multiplied by 100 thousand inhabitants to obtain the incidence rates for the analyzed years.

Only new cases were used for the analyses. Those who did not present a municipality of residence as a spatial locator, post-death cases, and relapse were disregarded as input to the system.

The epidemiological variables used in the descriptive analysis were: age group, clinical form, region of residence, area of residence, level of education, sex, ethnicity/skin color, Directly Observed Treatment (DOT), and closure status of the case. The categories of each variable prioritized the segment of the national notification form adopted by the Brazilian Unified Health System (SUS). The variables were categorized and expressed by absolute and relative frequencies. The Microsoft Office Excel 2017 software was used in the analysis.

### Temporal trend analysis

The temporal trend analysis was carried out using segmented log-linear regression models, using the Joinpoint Regression Program (version 4.2.0)^11^.

For the development of trends, six dependent variables were considered in each segment of analysis, namely: incidence of TB-HIV coinfection in Brazil; incidence of TB-HIV coinfection by Brazilian regions; by sex; by age group; and by area of residence; evolution of the case; and the years as an independent variable.

The Monte Carlo permutation test was used to select the best model by inflection points, applying 999 permutations and considering the highest coefficient of determination. The annual percent change (APC) and the increase in the average annual percent changes (AAPCs) were calculated for the entire period, when there was more than one significant inflection point^11^.

A trend was considered statistically significant when the APC showed p<0.05 and its 95% Confidence Interval (CI) did not include a zero value. Positive and significant APC and AAPC values indicated increasing trends; negative and significant, decreasing trends. Nonsignificant trends were described as stable regardless of APC and AAPC values^12^.

### Spatial analysis

The crude TB-HIV incidence coefficients were used in the analysis. However, the Local Bayesian Estimator was used to minimize the instability caused by the random fluctuation of the cases, smoothing the standardized coefficients by applying weighted averages and creating a second corrected coefficient. The Empirical Bayesian Rate illustrates the correction of the multiplicative rate equal to 100 thousand, considering the population at risk and the number of cases during the analyzed period^13,14^.

The global spatial autocorrelation between the crude coefficients was used to investigate whether the spatial distribution of the cases occurs randomly or if it follows a pattern in space. A spatial proximity matrix was developed, obtained using the contiguity criterion, adopting a significance level of 5%, and the Moran Global Index (I) was calculated, ranging from -1 to +1, which represents the expression of the spatial autocorrelation of the cases in the analyzed geographical space, to identify spatial clusters and risk areas. Values close to zero indicated spatial randomness; values between 0 and +1, positive spatial autocorrelation; and values between -1 and 0; negative spatial autocorrelation^15,16^.

Once global autocorrelation was identified, the occurrence of local autocorrelation was evaluated by calculating the Local Moran Index (Local Indicators of Spatial Association – LISA), which determines the dependence of local data on its neighbors. Once the statistical significance of the LISA was determined, areas that present a significantly different local correlation from the remaining of the data were identified, allowing the definition of spatial association patterns that may indicate the occurrence of spatial clusters, generating a four-quadrant scattering diagram^17^: Q1 (high/high) and Q2 (low/low), which indicate municipalities with values similar to those of the surrounding area and represent areas of agreement with aggregates of positive spatial association; Q3 (high/low) and Q4 (low/high), which indicate municipalities with different values and represent areas of transition with aggregates of negative spatial association^13^.

The analyses were performed using TerraView 4.2.2 and QGIS 3.4 software and the significant results were visually expressed in the form of choropleth maps, considering the local analyses of LISA Map and Moran Map.

## RESULTS

A total of 179,067 new cases of TB-HIV coinfection were reported in the period from 2001 to 2020 in Brazil. There was a predominance of men (71.0%), mixed-race (34.0%) skin color, age between 35 and 44 years (33.8%), with incomplete elementary school (37.7%), living in urban areas (70.0%), and in the Southeast region (46.2%) (Table 1).

**Table 1:** Epidemiological characteristics of the incidence of TB-HIV coinfection — Brazil (2001 to 2020).

Variables	Frequency (n)	Frequency (%)
Age group		
00–14	2,493	1.4
15–24	14,752	8.2
25–34	57,240	32.0
35–44	60,352	33.8
45–54	31,174	17.4
55–64	9,935	5.6
>65	2,872	1.6
Clinical form		
Pulmonar	118,338	66.0
Extrapulmonar	40,628	22.7
Pulmonary and extrapulmonary	20,094	11.3
Region of residence		
North	14,186	8.0
Northeast	32,346	18.0
Southeast	82,831	46.2
South	41,671	23.3
Midwest	7,036	4.0
Area of residence		
Urban	123,817	69.1
Rural	937	2.8
Ignored	50,264	28.1
Level of education		
Incomplete 1st to 4th grade of ES	17,910	10.0
Complete 4th grade of ES	6,920	3.9
Incomplete 5th to 8th grade of ES	42,549	23.8
Complete Elementary School	8,935	5.0
Incomplete High School	18,799	10.5
Complete High school	10,758	6.0
Incomplete higher education	2,936	1.6
Complete higher education	4,781	2.7
Illiterate	5,526	3.1
Ignored	59,953	42.5
Sex		
Men	127,063	71.0
Women	51,976	29.0
Ethnicity		
White	56,574	31.6
Black	24,063	13.4
Asian	888	0.5
Mixed-race	60,898	34.0
Indigenous	501	0.3
Ignored	36,137	20.2
Undergoing DOT		
Yes	36,523	20.4
No	59,838	33.4
Ignored	82,706	46.2
Closure status		
Cure	82,357	46.0
Dropout	34,567	19.3
Death	36,080	20.1
Transfer	15,845	8.9
Ignored	10,218	5.7

ES: elementary school; DOT: directly observed treatment.

Regarding the clinical form, a higher percentage of cases of the pulmonary form (66%) was observed. Regarding the variables associated with treatment, we observed a significant underreporting of data related to DOT, with 46.2% of the cases without recording this information. Among those registered, we observed that the majority of individuals did not undergo DOT (33.3%). In addition, regarding the closure status, we verified cure in most cases (46.0%). Nevertheless, it is worth highlighting the significant rates of death from TB (20.5%) and treatment dropout (17.2%) (Table 1).

**Figure 1:**
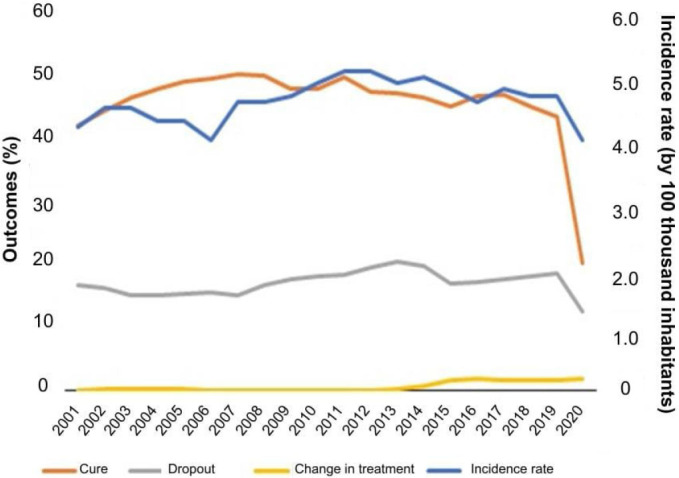
Evolution of the incidence rate (100 thousand inhabitants), proportion of cure, dropout, and change in treatment for TB-HIV coinfection — Brazil (2001 to 2020).

During the analyzed interval, the highest incidence of TB-HIV was observed in 2011 and 2012 (5.1 cases per 100 thousand inhabitants), followed by 2014 (5.0 cases per 100 thousand inhabitants). The highest percentage of cure was observed in 2007 (50.5%) and, during the period under study, this indicator did not reach the recommended values, showing only 46% of the individuals cured. Moreover, there was a higher dropout percentage in 2013 (20.6%) and changes in relation to treatment in 2016 (1.9%) (Figure 1).

In Chart 1 we present the trends in the crude incidence rates of TB-HIV coinfection. We identified a stable trend throughout Brazil, with an AAPC of 0.0% (95%CI -1.1 to 1.1; p=0.973). When analyzing the country’s regions individually, we observed increasing trends in the North (AAPC=7.1%; 95%CI 5.4–8.9; p≤0.001), Northeast (AAPC=6.0%; 95%CI 5.0–7.1; p≤0.001) and Midwest (AAPC=7.1%; 95%CI 5.4–8.9; p≤0.001); stable trend in the South (AAPC=-0.6%; 95%CI -1.6–0.4; p=0.232) and decreasing in the Southeast region (APC=-1.9%; 95%CI -2.3–1.5; p≤0.001). In addition, regarding the area of residence, increasing trends in rates were observed in urban, rural, and peri-urban areas, with a higher percentage increase in the latter (APC=6.4%; 95%CI 3.2–9.7; p≤0.001) (Chart 1).

**Chart 1:** Temporal trend of the incidence of TB-HIV coinfection in Brazil by region, area of residence, sex, and age group (2001 to 2020)

Characteristics		Years	Period by inflection points				Full period			
			APC	95%CI	p-value	Trend	AAPC	95%CI	p-value	Trend
TB-HIV incidence	Brazil	2001–2006	-0.6	-3.3; 2.0	0.608	Stable	0.0	-1.1; 1.1	0.973	Stable
		2006–2011	3.9*	0.3; 7.6	0.036	Increasing				
		2011–2020	-1.7*	-2.7; -0.7	0.002	Decreasing				
Regions of Brazil	North	2001–2014	10.8*	8.7; 12.9	<0.001	Increasing	7.1*	5.4; 8.9	<0.001	Increasing
		2014–2020	-0.5	-4.3; 3.4	0.776	Stable				
	Northeast	2001–2011	11.1*	9.3; 13.0	<0.001	Increasing	6.0*	5.0; 7.1	<0.001	Increasing
		2011–2020	0.6	-0.8; 2.0	0.359	Stable				
	Midwest	2001–2009	6.6*	4.1; 9.2	<0.001	Increasing	2.8*	1.7; 4.0	<0.001	Increasing
		2009–2020	0.2	-1.0; 1.4	0.739	Stable				
	Southeast	2001–2020	-1.9*	-2.3; 1.5	<0.001	Decreasing	-	-	-	-
	South	2001–2013	1.9*	0.8; 2.9	<0.001	Increasing	-0.6	-1.6; 0.4	0.232	Stable
		2013–2020	-4.7*	-6.8; -2.5	<0.001	Decreasing				
Area of residence	Urban	2001–2020	1.5*	0.8; 2.3	<0.001	Increasing	-	-	-	-
	Rural	2001–2020	5.3*	3.8; 6.9	<0.001	Increasing	-	-	-	-
	Periurban	2001–2020	6.4*	3.2; 9.7	<0.001	Increasing	-	-	-	-
Sex	Men	2001–2014	1.3*	0.6; 2.1	<0.001	Increasing	0.3	-0.5; 1.1	0.483	Stable
		2014–2020	-1.9	-4.2; 0.4	0.097	Stable				
	Women	2001–2012	2.3*	1.4; 3.2	<0.001	Increasing	0.0	-0.7; 0.8	0.893	Stable
		2012–2020	-3.0*	-4.3; -1.6	<0.001	Decreasing				
Age group	0 to 14	2001–2020	-5.6*	-6.9; -4.3	<0.001	Decreasing	-	-	-	-
	15 to 24	2001–2020	1.2*	0.5; 1.9	0.003	Increasing	-	-	-	-
	25 to 34	2001–2020	-1.2*	-1.6; -0.7	<0.001	Decreasing	-	-	-	-
	35 to 44	2001–2020	0.3	-0.8; 0.3	<0.315	Stable	-	-	-	-
	45 to 54	2001–2020	2.8*	1.6; 3.9	<0.001	Increasing	-	-	-	-
	55 to 64	2001–2020	5.3*	4.2; 6.4	<0.001	Increasing	-	-	-	-
	65 or over	2001–2020	5.3*	4.3; 6.3	<0.001	Increasing	-	-	-	-
Closure status	Cure	2001–2008	11.3	-12.6; 41.6	0.361	Stable	2.8	-	0.361	Stable
		2008–2020	-1.8*	-11.4; 9.0	0.719	Stable			0.719	Stable
	Dropout	2001–2020	4.3*	-2.3; 11.2	< 0.001	Increasing	-	-	0.193	Stable
	Death	2001–2020	1.9	-4.3; 8.4	1	Stable	-	-	0.540	Stable

APC: Annual Percentage Change; APCC: Average Annual Percent Changes;

* significant trend p<0.05.

When analyzing the trends in the incidence rates in the populations of men and women, we observed a stationarity process for both. Regarding the age group, a gradual increase is identified with increasing age, with a greater increase in incidence rates after 45 years of age, with the highest percent increases verified in the age groups of 55-64 years (APC=5.3%; 95%CI 4.2–6.4; p≤0.001) and among those over 65 years of age (APC=5.3%; 95%CI 4.3–6.3; p≤0.001). As for the outcome of the cases, we observed stable trends for cure, dropout, and death (Chart 1).

**Figure 2:**
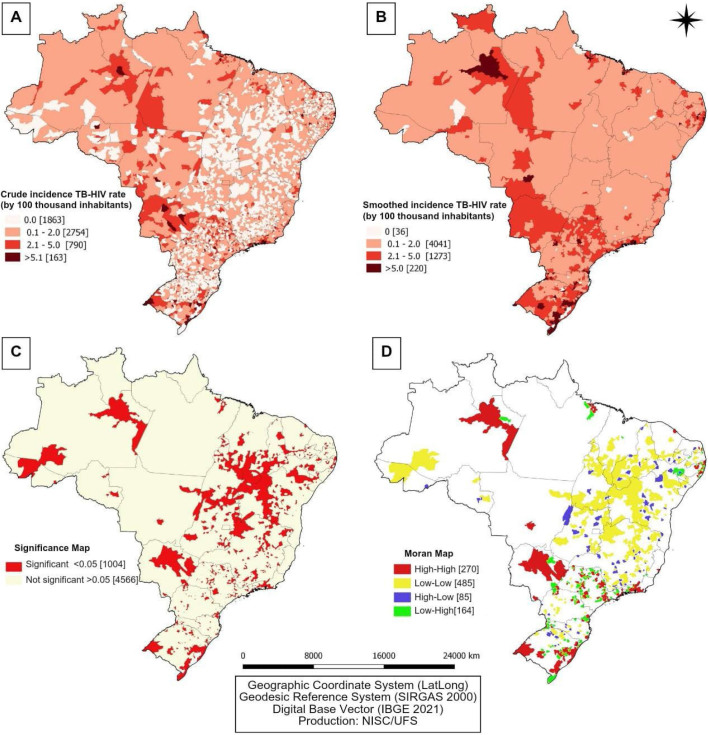
A. Spatial analysis of the incidence of TB-HIV coinfection in Brazil (2001 to 2020). B. Spatial analysis using the empirical Bayesian estimator of TB-HIV coinfection in Brazil (2001 to 2020). C. Spatial analysis considering the statistical significance of TB-HIV coinfection in the Brazilian territory between 2001 and 2020. D. Local Moran Index (LISA) of the incidence of TB-HIV — Brazil (2001-2020).

To identify areas with the highest concentration of TB-HIV cases, we evaluated the spatial distribution of coinfection among municipalities in Brazil and observed that the cases were widely distributed in the country. The comparison between the distribution of crude and smoothed rates is illustrated in Figures 2A and 2B, respectively. When analyzing the crude rates, we identified a dispersion of municipalities with a high incidence of TB-HIV in all Brazilian regions. However, considering the smoothed rates, there is a homogenization of cases and the existence of clusters formed by municipalities with a high incidence (5 or more cases per 100 thousand inhabitants) located in the states of Amazonas, Roraima, Pará, Pernambuco, Mato Grosso, Minas Gerais, São Paulo, Rio de Janeiro, Paraná, Santa Catarina, and Rio Grande do Sul.

Furthermore, we verified areas with significant local correlation, comprising 1,004 municipalities in all states of the country, with the exception of Amapá (Figure 2C). Likewise, we observed a positive and significant spatial autocorrelation of the high/high type, characterizing areas at high risk for the occurrence of TB-HIV coinfection, comprising 270 municipalities deemed priority, located mainly in the states of Amazonas, Mato Grosso, Mato Grosso do Sul, Rio de Janeiro, São Paulo, and Rio Grande do Sul (Figure 2D).

## DISCUSSION

The findings indicate that the incidence of TB-HIV coinfection in Brazil between 2001 and 2020 showed a stable trend. When analyzing the regions, there is an increasing trend toward the North, Northeast, and Midwest during the study period. We noticed that in the last decade there was a significant decrease in the incidence rate of TB-HIV. The reduction can be explained by advances in the healthcare system, in the diagnosis and early treatment of infections and, tragically, also by the decrease in the notification of cases of TB-HIV coinfection during the COVID-19 pandemic period.

Historically, the North and Northeast regions have a lower human development index when compared to other regions of the country. This factor may explain the growing trend of TB-HIV coinfection in the North and Northeast^9^. The worsening of social and economic conditions results in a significant degradation of living conditions, increasing vulnerability and, consequently, the risk of becoming ill with TB-HIV^18^.

Both in this study and in that by Cavalin^19^, there was an increase in the North, Northeast, and Midwest regions, while the South showed no significant difference. Unlike the others, the Southeast region maintained the decreased curve. Nevertheless, the analysis of regional trends must be carried out with caution due to the inequalities related to the recording of data in SINAN-TB, considering that increasing trends may reflect an improvement in the quality of the information system, and not necessarily an increase in incidence^18^.

It should also be considered that Brazilian regional differences reflect in different situations of diagnosis, treatment, and growth of coinfection in each region, a fact that can be explained by health policies implemented at different paces as well as supply capacity and discrepant access to services in each region. Historically, both the North and the Northeast have lower levels of development when compared to regions in the South and Southeast of the country, which may justify the increasing trend of coinfection in these regions.

The research conducted by Barbosa^20^, who analyzed TB-HIV coinfection in Northeastern Brazil, added factors that contribute to maintaining the rise in the incidence of TB-HIV coinfection in this region. The low cure rate, the high dropout rate, the occurrence of severe forms of extrapulmonary tuberculosis, and the high case fatality rate reflect the challenge in patient care and surveillance of cases of TB-HIV in the Northeast^20^.

The high level of absenteeism in data related to DOT is evident in this research: 46.2% ignored it and 33% did not undergo it, even though it was a goal of the national program against the elimination of TB. This fact raises questions about whether health information systems are being reliably fed. The cure, both in Barbosa^20^ and in this study, was not achieved, and underreporting also contributes to data incompleteness, reflecting in epidemiological surveillance systems results of organizational and structural problems, such as human failure, inadequate completion, and late notification, which reinforces the need for data improvement.

The clinical involvement of men coinfected with TB-HIV is a finding that is similar to several studies conducted in Brazil and is related to the fact that men are more likely to deny their vulnerability to diseases and to exempt themselves from responsibility for self-care, in addition to seeking healthcare services less.

In this study, the incidence of TB-HIV coinfection increased according to age group, which reflects a dangerous link between population aging and TB-HIV coinfection, making older adults more vulnerable to coinfection. Paiva et al.^21^ highlights that older adults do not consider themselves at risk of contracting the disease and, therefore, they do not usually use condoms during sexual intercourse because they consider this device suitable for use only by younger people^21^.

This study had limitations related to the technical-operational conditions of information systems, as it is known that the use of secondary sources, although official and widely used in scientific research, may present data incompleteness and divergent conditions, considering the low quality of the information system in some parts of the country. It should be noted that the ecological fallacy is one of the main biases of an epidemiological study, and complementarity with a cohort study would be important to advance this limitation. However, the analysis of the data was not compromised, and the relevance of the research problem can be assessed not only by the well-known weaknesses faced in the prevention and control of the disease, but mainly by the impacts caused to the population’s health.

The incidence of TB-HIV coinfection in Brazil has remained stable over the twenty years. When analyzed by region, it shows an increasing trend in the North, Northeast, and Midwest. The majority of cases of TB-HIV occurred predominantly in men, aged 35 to 44 years, of mixed-race ethnicity/skin color, living in urban areas, and with low level of education. In addition, the closure status did not meet the goals recommended for the TB-HIV eradication plan, representing only 46% with a cure outcome.

TB-HIV coinfection showed spatial correlation, pointing to a non-random distribution in space. Thus, we verified spatial clusters, with areas and neighbors showing high rates in 270 municipalities considered as priorities, located mainly in Amazonas, Mato Grosso, Mato Grosso do Sul, Rio de Janeiro, São Paulo, and Rio Grande do Sul.

Increasing trends in the incidence of TB-HIV coinfection were also observed among men and adults over the age of 40 years. Conversely, the cure rate has decreased over the years. The spatial analysis techniques made the study feasible and consisted of an important methodological tool for a better understanding of this problem and the definition of the main risk areas.

As implications for the collective health of the Brazilian population, it should be noted that the identification of the epidemiological profile of cases of TB-HIV coinfection in Brazil supports the development and implementation of public policies aimed at preventing this coinfection epidemic through a temporal and spatial approach.

It is suggested that new tools be developed to strengthen the adherence to treatment for TB-HIV coinfection based on the subjectivity of each individual and focused on social and economic determination, including the geographical location of risk areas and considering the pockets of poverty in large cities, precarious housing, existing comorbidities, and the use of alcohol and drugs. We emphasize that greater capacity in the diagnosis and treatment service must be concentrated where the needs of the individual, the family, and the community are greatest.
