# Supplementary material for: Surveillance of TB-HIV coinfection in Brazil: a space-time approach
Source: Rev Bras Epidemiol. 2024 Jul 15;27:e240037. doi: 10.1590/1980-549720240037 (PMC11251641; doi:10.1590/1980-549720240037)
Supplement: Translation

# Translation: Vigilância da coinfecção TB-HIV no Brasil: uma abordagem temporal e espacial

**DOI:** 10.1590/1980-549720240037.2

**Authors:** Beatriz Almeida Santos, Caíque Jordan Nunes Ribeiro, Allan Dantas dos Santos, Álvaro Francisco Lopes de Sousa, Thayane Santos Siqueira, Lucas Almeida Andrade, Adriano José dos Santos, Shirley Verônica Melo Almeida Lima

**Affiliations:** I Universidade Federal de Sergipe, Programa de Pós-Graduação em Enfermagem – São Cristóvão (SE), Brasil.; II Universidade Nova de Lisboa, Instituto de Higiene e Medicina Tropical – Lisboa, Portugal.; III Universidade Federal de Sergipe, Programa de Pós-Graduação em Ciências da Saúde – São Cristóvão (SE), Brasil.; IV Universidade Federal de Alagoas – Arapiraca (AL), Brasil.

**Keywords:** Tuberculose, HIV, Estudos de séries temporais, Análise espacial

## Abstract

**Objective:**

Identificar o padrão epidemiológico, espacial e temporal da coinfecção TB-HIV no Brasil durante o período de 2001 a 2020.

**Métodos:**

Estudo ecológico com técnicas de análise espacial e temporal. Incluiu os casos de coinfecção por TB-HIV registrados no Brasil entre 2001 e 2020. A análise de tendência temporal foi realizada por meio da regressão segmentada, através do *Joinpoint regression*. Para a análise espacial, foram calculados os índices de Moran e construídos mapas coropléticos através dos softwares *TerraView* e *QGIS*.

**Resultados:**

Observou-se tendência temporal estável das taxas de incidência da coinfecção por TB-HIV no Brasil durante o período analisado. Adicionalmente, foram identificadas áreas de alto risco para coinfecção localizadas em estados das regiões Norte, Sudeste, Sul e Centro-Peste.

**Conclusão:**

Houve estabilidade da incidência da coinfecção TB-HIV no Brasil nos últimos 20 anos e distribuição geográfica heterogênea das áreas de risco para o agravo.

## INTRODUÇÃO

A tuberculose (TB) permanece como um desafio à saúde pública mundial e tornou-se ainda mais desafiadora quando seus efeitos impactaram de forma devastadora as pessoas que vivem com o Vírus da Imunodeficiência Adquirida (HIV). A associação TB-HIV provoca importantes complicações clínicas e de adesão ao tratamento, evidenciando que indivíduos com HIV têm 28 vezes mais chances de contrair TB^1^. A elevada mortalidade, o aumento de casos de TB resistente aos medicamentos e a concentração da doença em populações socialmente vulneráveis estimularam a priorização do combate à doença, seja em nível global ou nacional^2^.

Ao longo dos anos, a TB permaneceu estigmatizada e assumiu diferentes conotações na história, com destaque para uma caracterização reemergente em alguns países da Europa Ocidental e da América^3,4^. No Brasil, se comporta como um grave problema de saúde pública envolvendo fatores multicêntricos, comportamentais e sociais. Globalmente, a TB causou 300 mil mortes entre as pessoas com HIV positivo. O Brasil é o único país das Américas com prioridade para ações de controle da coinfecção, pois representa 10,2% entre os diagnosticados com TB^5,6^.

Para viabilizar a resposta global, a Organização Mundial da Saúde (OMS) aprovou, na Assembleia Mundial da Saúde de 2014, a Estratégia End TB^5^ (pelo fim da tuberculose), que propõe uma mudança radical de paradigma na luta contra a TB, com o objetivo de erradicar a doença como problema de saúde pública: reduzir em 90% os casos de TB e em 95% as mortes por TB até 2035^5^.

As metas globais são sustentadas por três pilares:

1. Cuidados e prevenção integrados e centrados no paciente;
2. Políticas ousadas e sistemas de apoio e suporte aos afetados pela TB; e
3. Intensificação da inovação e da pesquisa^5^.

A emergência global da pandemia da COVID-19 culminou na reorganização de ações, serviços e sistemas de saúde em todo o mundo, e foram revertidos anos de progresso no controle da coinfecção TB-HIV^1^. O processo pandêmico colocou em risco a estratégia pelo fim da TB no Brasil e impactou diretamente os novos diagnósticos^2^.

A pandemia da COVID-19 exacerbou as desigualdades em saúde e favoreceu o negligenciamento da coinfecção TB-HIV desde o diagnóstico até o tratamento^2^. De 2012 a 2019, a proporção de casos novos de TB testados para HIV apresentou um aumento de aproximadamente 15%, chegando a 82,8%, especialmente em 2019. Todavia, em 2020 e 2021 houve um decréscimo importante^4^. É fato que durante o período de pandemia houve redução de testagens para o HIV em casos de TB.

Em países em desenvolvimento, como no Brasil, a desigualdade social representa terreno fértil para a disseminação da coinfecção ao considerar que a TB-HIV está associada a condições de vida, saúde e trabalho precárias. A OMS manifestou a preocupação com a possibilidade de comprometimento do progresso alcançado em relação à prevenção e ao tratamento da TB, ressaltando a importância de os programas nacionais da doença garantirem a continuidade das ações destinadas ao controle da enfermidade^4,6^.

Conhecer a distribuição temporal e espacial, assim como identificar o perfil das pessoas acometidas, contribui na orientação das políticas de controle da TB-HIV, direciona os esforços para as áreas com maior risco e orienta aquelas em que a situação operacional do programa de Controle Nacional da TB esteja aquém das metas estabelecidas. Nesse contexto, estudos dessa magnitude podem contribuir para o fortalecimento de novas investigações científicas e implementação de políticas públicas orientadas ao enfrentamento da coinfecção. Assim, objetiva-se neste trabalho identificar o padrão epidemiológico, espacial e temporal da coinfecção TB-HIV no Brasil durante o período de 2001 a 2020.

## MÉTODOS

### Tipo de estudo

Estudo ecológico com técnicas de análise espacial e tendência temporal. Foram incluídos todos os casos confirmados de coinfecção TB-HIV registrados no Brasil entre 2001 e 2020, considerando os meses de janeiro a dezembro em todos os anos, cujas unidades de análise e agregação foram os 5.570 municípios brasileiros. Temporalmente, foram coletados os dados notificados da coinfecção vinculados ao Sistema de Informação de Agravos de Notificação (SINAN), publicamente disponibilizados pelo Departamento de Informática do Sistema Único de Saúde do Brasil (DATASUS) em meio digital. Assim, foi dispensável a submissão ao comitê de ética.

### Área do estudo

O Brasil ocupa uma área de 8.510.345,540 milhões de km², o que equivale a quase 50% do território da América do Sul, e possui uma população total de 215,1 milhões de habitantes^7^, sendo o maior país da América Latina e o sexto maior contingente populacional do mundo. Delimitado pelo oceano Atlântico ao leste, tem um litoral de 7.491 km e faz fronteira com todos os outros países sul-americanos, exceto Chile e Equador. O Brasil está dividido em cinco regiões (Norte, Nordeste, Sudeste, Sul e Centro-Oeste) e 27 Unidades Federativas (UF)^8^. Conforme o relatório de Desenvolvimento Humano (2021/2022^9^), possui IDH de 0,754, portanto, encontra-se, na faixa de alto desenvolvimento, apesar de apresentar discrepâncias socioeconômicas importantes entre regiões^10^.

### Análise exploratória dos dados

Foram utilizados como variável de desfecho todos os novos casos de coinfecção TB-HIV registrados no Brasil entre 2001 e 2020 no SINAN. O número total de casos de TB-HIV foi dividido pela população de casos novos de TB e multiplicado por 100.000 habitantes para obtenção das taxas de incidência dos anos analisados.

Apenas os casos novos foram utilizados para as análises. Foram desconsiderados aqueles que não apresentaram município de residência como localizador espacial, casos pós-óbito e recidiva como entrada no sistema.

As variáveis epidemiológicas utilizadas na análise descritiva foram: faixa etária, forma clínica, região de residência, zona de residência, escolaridade, sexo, raça/cor, realização de Tratamento Diretamente Observado (TDO) e situação de encerramento do caso. As categorias de cada variável priorizaram o segmento da ficha de notificação nacional adotada no Sistema Único de Saúde (SUS). As variáveis foram categorizadas e expressas por frequências absolutas e relativas. O *software* Microsoft Office Excel 2017 foi o utilizado na análise.

### Análise da tendência temporal

A análise de tendência temporal foi realizada por meio de modelos de regressão log-linear segmentada, utilizando-se o *Joinpoint Regression Program* (versão 4.2.0)^11^.

Para o desenvolvimento de tendência, foram consideradas seis variáveis dependentes em cada segmento de análise: incidência de coinfecção TB-HIV no Brasil; incidência de coinfecção TB-HIV por regiões brasileiras; por sexo; por faixa etária e zona de residência; evolução do caso e os anos como variável independente.

O teste de permutação de Monte Carlo foi empregado para selecionar o melhor modelo por pontos de inflexão, aplicando-se 999 permutações e considerando-se o maior coeficiente de determinação. Foram calculados o incremento anual percentual (APC — *annual percent change*) e o incremento das médias percentuais anuais (AAPCs — *average annual percentage changes*) para todo o período, quando existia mais de um ponto de inflexão significativo^11^.

Foi considerada tendência estatisticamente significativa quando a APC apresentou p<0,05 e seu Intervalo de Confiança (IC) de 95% não incluía o valor zero. Os valores de APC e AAPC positivos e significativos indicaram tendências crescentes; os negativo e significativo, tendência decrescente. As tendências não significativas foram descritas como estáveis, independentemente dos valores de APC e AAPC^12^.

### Análise espacial

Os coeficientes brutos da incidência TB-HIV foram usados na análise. No entanto, empregou-se o Estimador Bayesiano Local para minimizar a instabilidade ocasionada pela flutuação aleatória dos casos, suavizando os coeficientes padronizados por aplicação de médias ponderadas e criando um segundo coeficiente corrigido. A Taxa Bayesiana Empírica ilustra a correção da taxa multiplicativa igual a 100.000, considerando-se a população em risco e o número de casos durante o período analisado^13,14^.

A autocorrelação espacial global entre os coeficientes brutos foi utilizada para investigar se a distribuição espacial dos casos ocorre de forma aleatória ou se segue algum padrão no espaço. Elaborou-se uma matriz de proximidade espacial, obtida pelo critério de contiguidade, adotando-se um nível de significância de 5%, e calculou-se o Índice Global de Moran (I), variando entre -1 e +1, que representa a expressão da autocorrelação espacial dos casos no espaço geográfico analisado, para identificar aglomerados espaciais e áreas de risco. Valores próximos a zero indicaram aleatoriedade espacial; valores entre 0 e +1 autocorrelação espacial positiva e entre -1 e 0 autocorrelação espacial negativa^15,16^.

Identificada a autocorrelação global, foi avaliada a ocorrência de autocorrelação local pelo cálculo do Índice de Moran Local (*Local Indicators of Spatial Association —* LISA), o qual determina a dependência de dados locais em relação a seus vizinhos. Uma vez determinada a significância estatística do LISA, foram identificadas áreas que apresentam correlação local significativamente diferente do resto dos dados, possibilitando a definição de padrões de associação espacial que podem indicar a ocorrência de aglomerados espaciais, gerando um diagrama de espalhamento de quatro quadrantes^17^: Q1 (alto/alto) e Q2 (baixo/baixo), que indicam municípios com valores semelhantes aos do entorno e representam áreas de concordância com agregados de associação espacial positiva; Q3 (alto/baixo) e Q4 (baixo/alto), que indicam municípios com valores diferentes e representam áreas de transição com agregados de associação espacial negativa^13^.

As análises foram realizadas por meio dos *softwares TerraView 4.2.2* e *QGIS 3.4* e os resultados significativos foram expressos visualmente em forma de mapas coropléticos, considerando as análises locais de Lisa Map e Moran Map.

## RESULTADOS

Foram notificados 179.067 novos casos de coinfecção TB-HIV no período de 2001 a 2020 no Brasil. Houve predomínio de indivíduos do sexo masculino (71,0%), pardos (34,0%), entre 35 e 44 anos de idade (33,8%), com ensino fundamental incompleto (37,7%), residentes em zona urbana (70,0%) e na região Sudeste (46,2%) (Tabela 1).

**Tabela 1:** Características epidemiológicas da incidência da coinfecção TB-HIV – Brasil (2001 a 2020).

Variáveis	Frequência (n)	Frequência (%)
Grupo etário		
00–14	2.493	1,4
15–24	14.752	8,2
25–34	57.240	32,0
35–44	60.352	33,8
45–54	31.174	17,4
55–64	9.935	5,6
>65	2.872	1,6
Forma		
Pulmonar	118.338	66,0
Extrapulmonar	40.628	22,7
Pulmonar e extrapulmonar	20.094	11,3
Região de residência		
Norte	14.186	8,0
Nordeste	32.346	18,0
Sudeste	82.831	46,2
Sul	41.671	23,3
Centro-Oeste	7.036	4,0
Zona de residência		
Urbana	123.817	69,1
Rural	937	2,8
Ignorado	50.264	28,1
Escolaridade		
1^a^ a 4^a^ série incompleta do EF	17.910	10,0
4^a^ série completa do EF	6.920	3,9
5^a^ a 8^a^ série incompleta do EF	42.549	23,8
Ensino fundamental completo	8.935	5,0
Ensino médio incompleto	18.799	10,5
Ensino médio completo	10.758	6,0
Educação superior incompleta	2.936	1,6
Educação superior completa	4.781	2,7
Analfabeto	5.526	3,1
Ignorado	59.953	42,5
Sexo		
Masculino	127.063	71,0
Feminino	51.976	29,0
Raça		
Branca	56.574	31,6
Preta	24.063	13,4
Amarela	888	0,5
Parda	60.898	34,0
Indígena	501	0,3
Ignorado	36.137	20,2
Realização de TDO		
Sim	36.523	20,4
Não	59.838	33,4
Ignorado	82.706	46,2
Situação de encerramento		
Cura	82.357	46,0
Abandono	34.567	19,3
Óbito	36.080	20,1
Transferência	15.845	8,9
Ignorado	10.218	5,7

EF: ensino fundamental; TDO: tratamento diretamente observado.

Quanto à forma clínica, observou-se maior percentual de casos da forma pulmonar (66%). Em relação às variáveis associadas ao tratamento, constatou-se importante subnotificação de dados referentes ao TDO, com 46,2% dos casos sem registro dessa informação. Dentre aqueles com registro, observou-se que a maior parte dos indivíduos não realizou o TDO (33,3%). Além disso, quanto à situação de encerramento, verificou-se a cura na maioria dos casos (46,0%). No entanto, é importante destacar os significativos percentuais de óbito por TB (20,5%) e abandono do tratamento (17,2%) (Tabela 1).

Durante o intervalo analisado, observou-se maior incidência por TB-HIV em 2011 e 2012 (5,1 casos por 100 mil habitantes), seguidos do ano de 2014 (5,0 casos por 100 mil habitantes). O maior percentual de cura foi observado em 2007 (50,5%) e, no período em estudo, esse indicador não alcançou os valores preconizados, apontando apenas 46% dos indivíduos curados. Adicionalmente, verificou-se maior percentual de abandono em 2013 (20,6%) e mudanças em relação ao tratamento em 2016 (1,9%) (Figura 1).

**Figura 1:** Evolução da taxa de incidência (100 mil habitantes), proporção de cura, abandono e mudança de tratamento por coinfecção TB-HIV – Brasil (2001 a 2020).

No Quadro 1 são apresentadas as tendências das taxas brutas de incidência da coinfecção por TB-HIV. Identificou-se uma tendência estável para todo o Brasil, que apresentou um AAPC de 0.0% (IC95% -1.1 a 1.1; p=0.973). Quando as regiões do país são analisadas individualmente, observam-se tendências crescentes no Norte (AAPC=7,1%; IC95% 5.4–8.9; p≤0.001), Nordeste (AAPC=6,0%; IC95% 5.0-7.1; p≤0.001) e Centro-Oeste (AAPC=7,1%; IC95% 5.4–8.9; p≤0.001); tendência estável no Sul (AAPC=-0,6%; IC95% -1.6–0.4; p=0.232) e decrescente na região Sudeste (APC=-1,9%; IC95% -2.3–1.5; p≤0.001). Além disso, quanto à zona de residência, foram visualizadas tendências crescentes das taxas nas zonas urbana, rural e periurbana, com maior incremento percentual nesta última (APC=6.4%; IC95% 3.2–9.7; p≤0.001) (Quadro 1).

**Quadro 1:** Tendência temporal da incidência da coinfecção TB-HIV no Brasil por região, zona de residência, sexo e faixa etária (2001 a 2020)

Características		Anos	Período por pontos de inflexão				Período completo			
			APC	IC95%	p-valor	Tendência	AAPC	IC95%	p-valor	Tendência
Incidência TB-HIV	Brasil	2001–2006	-0.6	-3.3; 2.0	0.608	Estável	0.0	-1.1; 1.1	0.973	Estável
		2006–2011	3.9*	0.3; 7.6	0.036	Crescente				
		2011–2020	-1.7*	-2.7; -0.7	0.002	Decrescente				
Regiões do Brasil	Norte	2001–2014	10.8*	8.7; 12.9	<0.001	Crescente	7.1*	5.4; 8.9	<0.001	Crescente
		2014–2020	-0.5	-4.3; 3.4	0.776	Estável				
	Nordeste	2001–2011	11.1*	9.3; 13.0	<0.001	Crescente	6.0*	5.0; 7.1	<0.001	Crescente
		2011–2020	0.6	-0.8; 2.0	0.359	Estável				
	Centro-Oeste	2001–2009	6.6*	4.1; 9.2	<0.001	Crescente	2.8*	1.7; 4.0	<0.001	Crescente
		2009–2020	0.2	-1.0; 1.4	0.739	Estável				
	Sudeste	2001–2020	-1.9*	-2.3; 1.5	<0.001	Decrescente	-	-	-	-
	Sul	2001–2013	1.9*	0.8; 2.9	<0.001	Crescente	-0.6	-1.6; 0.4	0.232	Estável
		2013–2020	-4.7*	-6.8; -2.5	<0.001	Decrescente				
Região de residência	Urbana	2001–2020	1.5*	0.8; 2.3	<0.001	Crescente	-	-	-	-
	Rural	2001–2020	5.3*	3.8; 6.9	<0.001	Crescente	-	-	-	-
	Periurbana	2001–2020	6.4*	3.2; 9.7	<0.001	Crescente	-	-	-	-
Sexo	Masculino	2001–2014	1.3*	0.6; 2.1	<0.001	Crescente	0.3	-0.5; 1.1	0.483	Estável
		2014–2020	-1.9	-4.2; 0.4	0.097	Estável				
	Feminino	2001–2012	2.3*	1.4; 3.2	<0.001	Crescente	0.0	-0.7; 0.8	0.893	Estável
		2012–2020	-3.0*	-4.3; -1.6	<0.001	Decrescente				
Faixa etária	0 a 14	2001–2020	-5.6*	-6.9; -4.3	<0.001	Decrescente	-	-	-	-
	15 a 24	2001–2020	1.2*	0.5; 1.9	0.003	Crescente	-	-	-	-
	25 a 34	2001–2020	-1.2*	-1.6; -0.7	<0.001	Decrescente	-	-	-	-
	35 a 44	2001–2020	0.3	-0.8; 0.3	<0.315	Estável	-	-	-	-
	45 a 54	2001–2020	2.8*	1.6; 3.9	<0.001	Crescente	-	-	-	-
	55 a 64	2001–2020	5.3*	4.2; 6.4	<0.001	Crescente	-	-	-	-
	65 ou mais	2001–2020	5.3*	4.3; 6.3	<0.001	Crescente	-	-	-	-
Situação de encerramento	Cura	2001–2008	11.3	-12.6; 41.6	0.361	Estável	2.8	-	0.361	Estável
		2008–2020	-1.8*	-11.4; 9.0	0.719	Estável			0.719	Estável
	Abandono	2001–2020	4.3*	-2,3; 11,2	< 0.001	Crescente	-	-	0.193	Estável
	Óbito	2001–2020	1.9	-4,3; 8,4	1	Estável	-	-	0.540	Estável

APC: Annual Percentage Change; APCC: Average Annual Percentage Changes;

* significative trend p<0.05.

Quando se analisam as tendências das taxas de incidência na população masculina e feminina, observa-se processo de estacionariedade para ambas. Já no tocante à faixa etária, é identificado aumento gradativo com o avanço da idade, com maior crescimento das taxas de incidência a partir de 45 anos, sendo os maiores incrementos percentuais visualizados nas faixas etárias de 55 a 64 anos (APC=5.3%; IC95% 4.2–6.4; p≤0.001) e entre maiores de 65 anos (APC=5.3%; IC95% 4.3–6.3; p≤0.001). Quanto ao desfecho dos casos, verificaram-se tendências estáveis para cura, abandono e óbito (Quadro 1).

Para identificar áreas com maior concentração de casos de TB-HIV, avaliou-se a distribuição espacial da coinfecção entre os municípios do Brasil e observou-se que os casos foram amplamente distribuídos no país. A comparação entre a distribuição das taxas brutas e suavizadas é visualizada nas Figuras 2A e 2B, respectivamente. Ao analisar as taxas brutas, identificou-se dispersão de municípios com alta incidência de TB-HIV em todas as regiões do Brasil. No entanto, considerando as taxas suavizadas, nota-se a homogeneização dos casos e a existência de aglomerados formados por municípios com elevada incidência (5 ou mais casos por 100 mil habitantes) localizados nos estados do Amazonas, Roraima, Pará, Pernambuco, Mato Grosso, Minas Gerais, São Paulo, Rio de Janeiro, Paraná, Santa Catarina e Rio Grande do Sul.

**Figura 2:** A: Análise espacial da incidência da coinfecção TB-HIV no Brasil (2001 a 2020). B: Análise espacial por meio do estimador bayesiano empírico da coinfecção TB-HIV no Brasil (2001 a 2020). C: Análise espacial considerando a significância estatística da coinfecção TB-HIV no território brasileiro entre 2001 e 2020. D: Índice de Moran Local (LISA) da incidência de TB-HIV – Brasil (2001-2020).

Adicionalmente, foram encontradas áreas que apresentam correlação local significativa, compreendendo 1.004 municípios em todos os estados do país, com exceção do Amapá (Figura 2C). Da mesma forma, foi encontrada autocorrelação espacial positiva e significativa do tipo alto/alto, caracterizando áreas de alto risco para a ocorrência da coinfecção TB-HIV, e que compreendem 270 municípios considerados prioritários, localizados principalmente nos estados do Amazonas, Mato Grosso, Mato Grosso do Sul, Rio de Janeiro, São Paulo e Rio Grande do Sul (Figura 2D).

## DISCUSSÃO

Os achados apontam que a incidência de coinfecção TB-HIV no Brasil entre 2001 e 2020 apresentou tendência estável. Quando analisadas as regiões, destaca-se tendência crescente para Norte, Nordeste e Centro-Oeste durante o período de estudo. É possível notar que na última década houve decréscimo significativo na taxa de incidência de TB-HIV. A redução pode ser explicada por avanços no sistema de saúde, no diagnóstico e tratamento precoce das infecções e, tragicamente, também pela diminuição da notificação dos casos de coinfecção TB-HIV no período de pandemia da COVID-19.

Historicamente as regiões Norte e Nordeste apresentam índice de desenvolvimento humano menor quando comparadas às demais regiões do país. Esse fator pode explicar a tendência crescente de coinfecção TB-HIV no Norte e Nordeste^9^. O agravamento das condições sociais e econômicas resulta em uma degradação significativa das condições de vida, aumentando a vulnerabilidade e, consequentemente, o risco de adoecimento por TB-HIV^18^.

Tanto neste estudo quanto no de Cavalin^19^ houve crescimento nas regiões Norte, Nordeste e Centro-Oeste, ao passo que a Sul não apresentou diferença significativa. Ao contrário das demais, a região Sudeste manteve a curva decrescente. Não obstante, a análise das tendências regionais deve ser feita com cautela devido às desigualdades referentes ao registro dos dados no SINAN-TB, visto que as tendências de crescimento podem refletir uma melhoria na qualidade do sistema de informação, e não necessariamente um amento na incidência^18^.

Há, ainda, que se considerar que as diferenças regionais brasileiras manifestam situações divergentes de diagnóstico, tratamento e crescimento da coinfecção em cada região, fato que pode ser explicado por políticas de saúde implementadas com velocidades diferentes, bem como capacidade de oferta e discrepante acesso a serviços em cada região. Tanto o Norte quanto o Nordeste, historicamente, apresentam níveis de desenvolvimento mais baixos quando comparados a regiões do Sul e Sudeste do país, o que pode justificar a tendência crescente da coinfecção nessas regiões.

A pesquisa de Barbosa^20^, que analisou a coinfecção TB-HIV no Nordeste do Brasil, acrescentou fatores que contribuem para manter a ascensão da incidência de coinfecção TB-HIV nessa região. O baixo percentual de cura, o elevado percentual de abandono, a ocorrência de formas graves de tuberculose extrapulmonar e a elevada taxa de letalidade refletem o desafio na assistência ao paciente e na vigilância dos casos de TB-HIV no Nordeste^20^.

Fica nítido nesta pesquisa o alto nível de absenteísmo de dados relacionados ao TDO: 46,2% o ignoraram e 33% não o realizaram, mesmo sendo uma meta do programa nacional contra a eliminação da TB. Esse fato levanta questionamentos sobre se os sistemas de informações em saúde estão sendo alimentados com fidedignidade. A cura, tanto em Barbosa^20^ quanto neste estudo, não foi alcançada, e a subnotificação também contribui para incompletude dos dados, refletindo nos sistemas de vigilância epidemiológica resultados de problemas organizacionais e estruturais, como falha humana, preenchimento inadequado e notificação tardia, o que reforça a necessidade de melhoria dos dados.

O maior acometimento a indivíduos do sexo masculino coinfectados por TB-HIV é um achado que se assemelha a diversos estudos realizados no Brasil e está relacionado ao fato de que homens são mais propensos a negar sua vulnerabilidade a doenças e se eximirem da responsabilidade pelo autocuidado, além de procurarem menos os serviços de saúde.

Neste estudo a incidência da coinfecção TB-HIV aumentou de acordo com a faixa etária, o que reflete um elo perigoso entre o envelhecimento populacional e a coinfecção TB-HIV, tornando os idosos mais vulneráveis à coinfecção. Paiva et al.^21^ destaca que a população idosa não se percebe em risco de contrair a doença, por conseguinte, não costuma utilizar o preservativo nas relações sexuais por considerar esse dispositivo de uso adequado apenas para os mais jovens^21^.

Este estudo apresentou limitações relacionadas às condições técnico-operacionais dos sistemas de informação, pois sabe-se que o uso de fontes secundárias, embora oficial e amplamente utilizadas em trabalhos científicos, pode apresentar incompletude de dados e condições divergentes, considerando a baixa qualidade do sistema de informação em alguns locais do país. Salienta-se que a falácia ecológica é um dos principais vieses de um estudo epidemiológico, sendo que a complementariedade com um estudo de coorte seria importante para avançar nessa limitação. Entretanto, a análise dos dados não foi comprometida, e a relevância da problemática de pesquisa pode ser aquilatada não somente pelas conhecidas fragilidades enfrentadas na prevenção e no controle da doença, mas, principalmente, pelos impactos causados à saúde da população.

A incidência da coinfecção TB-HIV no Brasil tem se mantido estável ao longo dos vinte anos. Quando analisada por região, apresenta tendência crescente no Norte, Nordeste e Centro-Oeste. A maioria dos casos de TB-HIV ocorreu predominantemente em indivíduos do sexo masculino, de 35 a 44 anos, de raça/cor parda, residentes na zona urbana e com baixa escolaridade. Ademais, a situação de encerramento não atingiu as metas preconizadas para o plano de erradicação da TB-HIV, representando apenas 46% com desfecho de cura.

A coinfecção TB-HIV apresentou correlação espacial, apontando para uma distribuição não aleatória no espaço. Assim, foram percebidos aglomerados espaciais, com áreas e vizinhos apresentando altas taxas em 270 municípios tidos como prioritários, localizados principalmente no Amazonas, Mato Grosso, Mato Grosso do Sul, Rio de Janeiro, São Paulo e Rio Grande do Sul.

Tendências crescentes da incidência de coinfecção TB-HIV também foram observadas entre homens e adultos com idade superior a 40 anos. Já o percentual de cura tem decrescido ao longo dos anos. As técnicas de análise espacial tornaram o estudo viável e configuraram-se como uma importante ferramenta metodológica para um melhor conhecimento desse agravo e definição das principais áreas de risco.

Como implicações para a saúde coletiva da população brasileira, salienta-se que a identificação do perfil epidemiológico dos casos de coinfecção TB-HIV no Brasil subsidia a elaboração e implementação de políticas públicas direcionadas à prevenção dessa epidemia da coinfecção por meio de uma abordagem temporal e espacial.

Sugere-se o desenvolvimento de novas ferramentas que fortaleçam a adesão ao tratamento da coinfecção TB-HIV com base na subjetividade de cada indivíduo e foco na determinação social e econômica, incluindo a localização geográfica de áreas de risco e considerando os bolsões de pobreza das grandes cidades, a moradia precária, as comorbidades existentes e o uso de álcool e drogas. Realça-se que a maior capacidade no serviço de diagnóstico e tratamento deve estar concentrada onde as necessidades do indivíduo, da família e da comunidade são maiores.
